# Diet Versus Exercise in Cardiometabolic Disease: Are Two Always Better Than One?

**DOI:** 10.1007/s11886-026-02419-8

**Published:** 2026-09-15

**Authors:** Jingyeong Jo, Kyuwan Lee

**Affiliations:** Department of Kinesiology and Sports Studies, Ewha Womans University, Seoul, Korea

**Keywords:** Cardiometabolic disease, Dietary intervention, Exercise intervention, Lifestyle modification

## Abstract

**Purpose of Review:**

While studies have established the independent benefits of diet and exercise, comparatively few have assessed these modalities side by side, evaluating diet-only, exercise-only, and combined interventions under comparable conditions. This review focuses on randomized trials and meta-analyses that provide such direct comparisons, emphasizing outcome-specific differences and the circumstances in which combining both modalities yields added benefit.

**Recent Findings:**

Evidence indicates that the superiority of combined diet and exercise interventions is outcome-dependent rather than universal. Dietary interventions generally produce improvements in body weight, cholesterol, and fasting glucose, whereas exercise uniquely enhances cardiorespiratory fitness, muscular strength, endothelial function, and physical function. For cardiometabolic outcomes including insulin sensitivity and visceral adiposity, combined interventions frequently provide additive or synergistic benefits. However, for functional outcomes such as VO₂peak and muscular performance, exercise remains the primary determinant, and adding dietary modification confers limited additional benefit. Similarly, blood pressure responses to combined interventions vary widely and do not consistently exceed those produced by nutrient-focused dietary patterns alone.

**Summary:**

Diet and exercise exert complementary but nonredundant effects across cardiometabolic pathways, and their combined advantages depend on the specific clinical outcome targeted. While integrated lifestyle interventions offer the broadest overall benefit, implementing both simultaneously is often constrained by practical and behavioral barriers. A patient-centered, outcome-specific strategy prioritizing diet or exercise based on the individual’s metabolic profile, functional status, and readiness may support adherence and provide a scalable path toward multimodal intervention. Such tailored sequencing has the potential to optimize cardiometabolic health and extend healthspan.

## Introduction

Despite major advances in medical therapy, a substantial residual risk of cardiometabolic disease persists, mainly attributable to lifestyle factors such as diet and exercise. Both lifestyle modalities improve a broad range of cardiometabolic risk factors, including blood pressure, lipid metabolism, glycemic regulation, and body composition [1, 2]. In general, exercise interventions utilizing aerobic and resistance training enhance cardiorespiratory fitness and muscular strength while also promoting improvements in endothelial function, skeletal muscle insulin sensitivity, and mitochondrial biogenesis [3, 4]. Dietary interventions such as the Mediterranean and whole-food plant-based diets optimize nutrient quality by increasing fiber, phytochemical density, and unsaturated fats, while reducing sodium, added sugars, and pro-inflammatory food components. These patterns are especially effective in reducing LDL-C, improving glycemic control, and facilitating weight loss.

Although each intervention yields significant health benefits independently, combining diet and exercise may or may not result in additive or synergistic effects, depending on the outcome assessed. For instance, dietary interventions are effective in reducing adiposity and improving lipid profiles, whereas exercise is frequently associated with preservation of lean mass and gains in physical function, although these patterns can vary depending on the population and intervention characteristics [5]. Importantly, exercise and dietary interventions each present unique barriers to implementation. For example, adherence to exercise interventions is often constrained by limited space, fatigue, or time demands, whereas dietary intervention is often hindered by entrenched habits or taste preferences. Understanding the relative contributions of diet and exercise, and knowing when and for whom each modality is most appropriate, is key to improving clinical effectiveness and ensuring long-term sustainability.

This review summarizes current evidence on the independent and combined effects of diet and exercise on cardiometabolic risk, drawing especially on randomized trials and meta-analyses that directly compare these lifestyle interventions. It incorporates clinical and implementation studies to delineate domain-specific benefits, identify barriers to adherence, and propose practical strategies for real-world integration.

## Energy Restriction Strategies and Their Influence on Cardiometabolic Risk

**Fig. 1:**
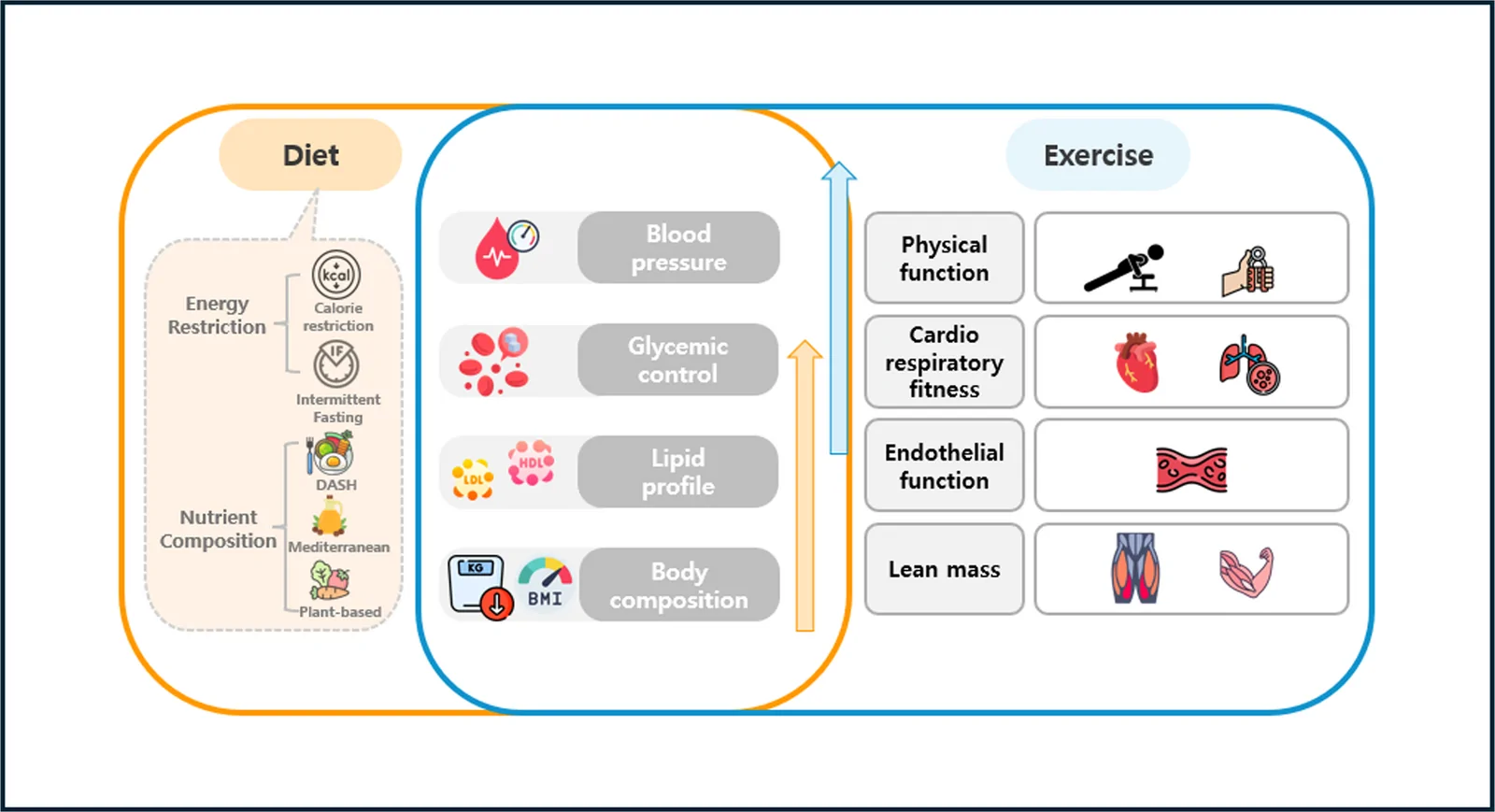
Synergistic and independent effects of exercise and nutrition on cardiometabolic health

Dietary intervention by itself can reduce body weight, improve glycemic control, and favorably influence blood pressure and lipid metabolism. These benefits arise from two main domains: energy restriction that lowers caloric intake or eating time window, and nutrient composition that prioritizes macronutrient quality and bioactive compounds (Fig. 1). In the context of energy restriction, two distinct approaches are commonly described: time-restricted eating (TRE) and total caloric reduction (CR). TRE involves limiting food consumption to specific hours of the day, thereby aligning circadian rhythms with metabolic processes [6]. A related approach, alternate-day fasting (ADF), alternates between days of energy restriction and days of normal eating and represents another form of energy restriction. In contrast, CR represents the traditional approach of limiting total caloric intake while maintaining sufficient nutrient intake.

Evidence comparing weight loss efficacy demonstrates that all energy restriction strategies consistently induce weight reduction compared to ad-libitum feeding. A recent meta-analysis results reveal that ADF achieves the greatest effect (−3.42 kg), followed by TRE (−2.25 kg), short-term fasting (−1.87 kg), and CR (−1.59 kg) [6]. TRE demonstrates particular efficacy not only in weight reduction but also in improving fasting glucose and insulin sensitivity. In this study, various TRE protocols achieved a mean fasting glucose reduction of −2.98 mg/dL (95% CI −4.70 to −1.26, *p* < 0.001). Similarly, Sun et al. demonstrated that TRE with 7–9 h eating windows resulted in a −2.82 mg/dL reduction compared to unrestricted eating (95% CI −4.51 to −1.12, *p* = 0.001) [7]. These consistent results suggest that TRE is an effective strategy for managing cardiometabolic risk factors. CR also significantly improves insulin sensitivity, reducing fasting insulin. These improvements are likely mediated by reductions in visceral fat (*r* = − 0.51; *p* < 0.01) and subcutaneous fat (*r* = − 0.32; *p* < 0.05), which are associated with enhanced metabolic regulation and lower inflammatory burden.

Lipid metabolism represents another critical domain for energy restriction strategies, with meaningful effects reported across various studies. CR significantly reduces LDL-C (−22.03 mg/dL) and total cholesterol (−12.72 mg/dL) [8], while TRE demonstrates confirmed HDL-C increases (MD = + 0.03 mmol/L, *p* = 0.010) [7]. Additionally, the 10-week ADF intervention reduced total cholesterol (− 37 mg/dL), LDL-C (− 30 mg/dL), and triglycerides (− 34 mg/dL), corresponding to decreases of approximately 21%, 25%, and 32%, respectively [9]. However, 6-month long-term studies revealed no significant between-group differences in total cholesterol (−19 vs. −18 mg/dL) and LDL-C (−11 vs. −10 mg/dL) reductions between ADF and CR [10, 11]. Short-term trials consistently show substantial lipid improvements, but these effects diminish in longer interventions, highlighting the importance of considering intervention duration when evaluating expected benefits.

The effects of energy restriction approaches on blood pressure show inconsistent results across studies. Sun et al. (2024) found that in overweight and obese adults, TRE showed lower systolic blood pressure reduction benefits than continuous CR (SMD = 0.21; 95% CI 0.05–0.36; *p* = 0.008) [7]. TRE reduced systolic blood pressure by −4.15 mmHg (*p* < 0.0001) despite no significant changes in diastolic blood pressure. Diastolic blood pressure reduction was observed only in studies lasting 12 weeks or longer (WMD = −1.916 mmHg; 95% CI −3.037 to −0.794; *p* = 0.001) [12]. Conversely, ADF showed no significant differences in blood pressure reduction compared to moderate CR in 8-week interventions [13], and CR also showed non-significant reductions in both systolic (−2.45 mmHg; 95% CI −5.24 to 0.35) and diastolic blood pressure (−0.65 mmHg; 95% CI −2.03 to 0.72). Thus, energy restriction can be an effective means of improving cardiometabolic risk factors, yet its outcomes are highly contingent on the specific approach employed and the length of the intervention.

## Nutrient Quality–Focused Dietary Approaches and Their Cardiometabolic Effects

The Dietary Approaches to Stop Hypertension (DASH) diet emphasizes sodium reduction and potassium enhancement to restore electrolyte balance and control blood pressure. The blood pressure lowering effects of the DASH diet have been well established for decades, with randomized trials demonstrating significant reductions of −5.5 mmHg in systolic and − 3.0 mmHg in diastolic blood pressure (*p* < 0.001), and greater effects in hypertensive patients (systolic − 11.4 mmHg; diastolic − 5.5 mmHg, *p* < 0.001) [14]. These effects were further confirmed in a large-scale meta-analysis including 41 clinical trials (8,416 participants), where the DASH diet showed powerful blood pressure reduction of −5.53mmHg and − 5.47mmHg, respectively, while the Mediterranean diet showed relatively limited effects of −0.95mmHg [15]. While the Mediterranean diet shows limited blood pressure reduction effects, an umbrella review including 29 meta-analyses (12.8 million participants) showed that Mediterranean diet significantly reduced all-cause mortality (RR = 0.90, 95% CI: 0.86–0.94, *p* < 0.001), cardiovascular disease (RR = 0.81, 95% CI: 0.75–0.87, *p* < 0.001), and coronary heart disease (RR = 0.82, 95% CI: 0.76–0.88, *p* < 0.001) [16].

Contrary to blood pressure effects, mixed results were reported for glucose and lipid metabolism. Mediterranean diet and plant-based diet significantly reduced HbA1c by 0.3–0.4% in type 2 diabetes patients [17]. However, while meta-analyses confirmed significant fasting glucose reduction [18, 19], others showed no effects [16]. For lipid metabolism, total cholesterol reductions of −0.14 to −0.20 mmol/L were consistently reported, while most studies showed no significant changes in LDL cholesterol. HDL cholesterol showed high heterogeneity with mixed results, and triglycerides varied from large reductions (−0.29 mmol/L) [20] to small reductions (−0.09 mmol/L) [21]. Meta-analysis showed Mediterranean diet adherence resulted in an average − 1.75 kg weight loss (95% CI: −2.86 to −0.64, *p* < 0.05) [22, 23], with other analyses reporting modest weight losses within the − 0.29 to −2.24 kg range [18, 20]. BMI and waist circumference also showed reductions but were limited to −0.29 to −0.61 kg/m² and − 0.42 to −1.93 cm, respectively [18, 19, 24].

## Synergistic Benefits of Energy Restriction Approaches Combined with Exercise

A substantial body of evidence indicates that the combination of exercise and energy restriction yields greater effects on weight loss and body composition compared with single interventions (Table 1). Recent meta-analysis of overweight or obese adults demonstrated that combined TRE and exercise interventions showed significant additional reductions compared to TRE alone in body weight (−3.03 kg), BMI (−1.12 kg/m²), body fat mass (SMD: −0.72), and waist circumference (−2.51 cm) [25]. Recent studies also demonstrated that high-intensity interval training (HIIT) combined with TRE induced greater weight loss than TRE alone (−3.2 vs. −2.1 kg, *p* < 0.05) [26]. Importantly, exercise also plays a critical role in preserving lean mass during weight reduction. This is supported by randomized interventions directly comparing diet and exercise. Diet alone produced substantial reductions in body weight (− 7.1 kg, *p* < 0.0001) and body fat (− 6.1 kg, *p* < 0.0001) but resulted in significant lean mass loss (− 0.8 kg, *p* < 0.0001). In contrast, exercise alone led to modest decreases in body weight (− 2.0 kg, *p* = 0.03) and body fat (− 2.1 kg, *p* < 0.0001) while increasing lean mass (+ 0.3 kg, *p* < 0.0001). The combined diet and exercise intervention produced the greatest reductions in body weight (− 8.9 kg, *p* < 0.0001) and body fat (− 8.2 kg, *p* < 0.0001) while limiting lean mass loss to − 0.4 kg (*p* = 0.15), demonstrating superior preservation compared with diet alone [27].

**Table 1:** Summary of randomized controlled trials and meta-analyses comparing exercise and dietary interventions

Author, year, country	Study type	Population characteristics	Intervention types	Comparators (control group)	Key results	Adherence	Duration
Blumenthal et al., USA [31]	Three-arm randomized controlled trial; 144 participants; DASH alone(DASH-A) = 46, DASH and Weight Management (DASH-WM) = 49, Control = 49	Overweight or obese adults ≥ 35 years with elevated BP (SBP 130–159 mmHg or DBP 85–99 mmHg); Mean age ≈ 52 years; 67% female; BMI 25–40 kg/m²; Sedentary and unmedicated for hypertension	DASH-A: Instruction to follow DASH diet only; Weekly small-group nutrition counseling, isocaloric meals to maintain weight; No exercise and no intentional weight loss DASH-WM: DASH diet and caloric restriction (− 500 kcal/day), weekly DASH counseling and cognitive-behavioral weight-loss sessions; Supervised aerobic exercise, 3 sessions/week at 70–85% HR reserve, exercise consisted of 10-min warm-up, 30-min brisk walking/jogging or cycling, 5-min cool-down Control group: Usual diet controls; asked to maintain typical diet and physical activity; Biweekly monitoring only; no structured intervention	Usual diet controls; asked to maintain typical diet and physical activity, biweekly monitoring only; no structured intervention	DASH-A: Significant blood pressure reductions(clinic SBP − 11.2 mmHg, DBP − 7.5 mmHg, *p* < 0.001); Improved arterial stiffness (lower pulse wave velocity, *p* = 0.001); No weight loss (−0.3 kg, NS); No cardiovascular fitness improvement (peak VO₂ −1.2%); No baroreflex sensitivity or LV mass changes DASH-WM: Greatest blood pressure benefits(clinic SBP − 16.1 mmHg, DBP − 9.9 mmHg, *p* < 0.001), superior to DASH alone for both SBP (*p* = 0.02) and DBP (*p* = 0.048); Significant weight loss (−8.7 kg, *p* < 0.001); Major cardiovascular fitness gain (peak VO₂ +19%, *p* < 0.001); Enhanced baroreflex sensitivity (+ 33%, *p* = 0.01 vs. DASH alone); Reduced LV mass index relative to DASH alone (*p* = 0.02); Greater arterial stiffness improvement	Median exercise class attendance: 90% (38 of 42 sessions) DASH diet class attendance: ≈ 92% for both DASH-A and DASH-WM	4 months
Weiss et al., USA [29]	Three-arm randomized controlled trial, 52 overweight, sedentary adults; Calorie Restriction (CR) = 17, Exercise (EX) = 16, Calorie Restriction and Exercise (CREX) = 19	Overweight, sedentary adults at risk for cardiovascular disease (no diagnosed CVD, BP below hypertension criteria); Mean age ≈ 57 ± 5 years; BMI 25.0–29.9 kg/m²	CR only: Dietary energy intake reduced by ~ 20%; Food diaries used for individualized guidance, encouraged lower energy-density foods and portion reduction, macronutrient distribution within recommended ranges; No exercise increase allowed EX Only: Endurance exercise; Energy expenditure increased by ~ 20% of baseline total energy expenditure, intensity encouraged at moderate to high (mean 77% HRmax), ~ 8 sessions/week, ~ 7.4 h/week; No dietary modification allowed CREX: 10% energy deficit from diet and 10% from exercise, ~ 6 sessions/week, ~ 4.4 h/week; Same dietary counseling as CR group with reduced volume; Total targeted deficit 20%	Comparison between CR, EX, CREX; Weight loss matched intentionally across groups (≈ 7%) to isolate method-specific effects	CR group: Total cholesterol and triglycerides decreased relative to Control (*p* < 0.05); Glucose declined (*p* < 0.05); Marked reductions in body weight and body fat (*p* < 0.001); No VO₂max improvement; Preserved fat-free mass better than CREX EX group: VO₂max increased (+ 22%, *p* < 0.001); Cholesterol improved similarly to CR; Body weight and fat mass decreased (*p* < 0.001); Unique preservation of fat-free mass; No additional metabolic benefits vs. CR alone CREX group: Greatest triglyceride reduction (−32 mg/dL, *p* < 0.001); Moderate VO₂max increase (+ 11%, *p* < 0.001); Similar cholesterol and glucose changes as other groups; Some fat-free mass loss	CR group: Maintained adherence through continuous dietary counseling based on 3-day food diaries EX group: Average of 8 sessions per week for a total of 7.4 h per week CREX group: Average of 6 sessions per week for a total of 4.4 h per week	Weight loss over 12–14 weeks and 2 weeks weight stabilization
Lee et al., South Korea [38]	Three-arm randomized control trial; 85 participants; Control = 28, Diet = 30, Diet and Exercise = 27	Adults ≥ 20 years with prehypertension or stage I hypertension; Mean age 45.1 ± 13 years; 78% male; 82% overweight or obese (BMI ≈ 26 kg/m²)	Diet only: Korean-modified DASH diet; Replace white rice with whole grains(barley), prefer chicken or fish over red meat, increase nuts, avoid high-fructose foods, and reduce salt by avoiding pickles or condiments Diet and Exercise: same diet and home-based exercise (pedometer, diary, and exercise DVD: 3 circuits of 5–7 core & resistance exercises; >10,000 steps/day; weekly phone check-in)	No structured education on diet or exercise	Diet group: no significant changes in office BP or ambulatory BP compared with Control; waist circumference decreased compared with Control; No significant changes in metabolic outcomes or functional outcomes Diet and Exercise group: significant reduction in daytime SBP (− 5.4 mmHg vs. Control + 0.8 mmHg, *p* = 0.003); Significant reduction in daytime DBP (− 4.1 mmHg vs. Control − 0.1 mmHg, *p* = 0.004); Significant reductions in 24-hour SBP(*p* = 0.001) and DBP(*p* = 0.009); No significant change in office BP compared with Control; Waist circumference significantly reduced compared with Control(*p* = 0.021)	Exercise diary return rate approximately 70% in Diet and exercise group Dietary adherence modest (self-reported improvement, urinary sodium not significantly changed in D group)	8 weeks
Haganes et al., Norway [30]	Four-arm randomized controlled trial; 131 participants; TRE(Time-Restricted Eating) = 33, HIIT(High-Intensity Interval Training) = 33, TREHIIT(Time-restricted eating and high intensity interval training) = 32, Control = 33	Overweight or obesity women; Mean age ≈ 36 years(aged 18–45 years); BMI ≥ 27 kg/m²; Normoglycemic at baseline; Able to perform treadmill or cycling exercise; No diabetes or cardiovascular disease	TRE: Self-selected ≤ 10-hour eating window per day with ad libitum intake; No macronutrient restriction; Recommended last meal before 20:00 HIIT: Supervised high-intensity interval training; Three sessions per week, two sessions of 4 × 4 min at 90–95% HRmax with 3-min recovery, one session of 10 × 1 min at ≥ 90% HRmax with 1-min recovery TREHIIT: TRE protocol and HIIT protocol combined Control group: No structured diet or exercise modification; asked to maintain habitual behaviors	Non-intervention control maintaining usual diet, physical activity, and daily routines	TRE group: No improvement in glucose tolerance(*p* = 0.36); Significant nocturnal glucose reduction(−0.4 mmol/L, *p* < 0.001); Moderate weight loss (−2.1 kg, *p* < 0.001) with visceral fat reduction(−8.0 cm², *p* = 0.002); Muscle mass loss (−0.4 kg, *p* = 0.039); No cardiovascular fitness or lipid improvements HIIT(High-Intensity Interval Training) group: Marginal glucose tolerance improvement(peak OGTT − 0.6 mmol/L, *p* = 0.047); Moderate weight loss(−1.7 kg, *p* = 0.005) with significant visceral fat reduction(−9.2 cm², *p* < 0.001); Major cardiorespiratory fitness gain(+ 3 mL/kg/min VO₂peak, *p* < 0.001); No blood pressure or lipid benefits TREHIIT group: Greatest weight loss (−3.6 kg, *p* < 0.001) and visceral fat reduction (−16.8 cm², *p* < 0.001); Improved glycemic control (HbA1c −1.1 mmol/mol, *p* = 0.008); Significant cardiorespiratory fitness improvement (+ 3.4 mL/kg/min, *p* < 0.001); HDL reduction (*p* = 0.016); Combined benefits of both interventions with additive effects on body composition	Not reported	7 weeks
Sampaio et al., Portugal [36]	Four-arm quasi-experimental trial; 87 participants (73 with complete blood data) Control = 19, Multicomponent Training (MT) = 20, Sustainable Healthy Diet (SHD) = 26, SHD and MT = 22	Community-dwelling adults ≥ 65 years; mean age ≈ 72 years ~ 74% female; Overweight to class I obesity range (BMI ≈ 28–31 kg/m²); Independent mobility and no major cardiometabolic or musculoskeletal contraindications	Diet Only: Mediterranean and plant-based sustainable diet; including walnuts, pulses, extra-virgin olive oil, and oily fish; Three group nutrition sessions with a Mediterranean culinary workshop and one nutrition phone interview Exercise Only: Multicomponent training; Supervised 50-minute group sessions three times per week including warm-up mobility and stretching, aerobic conditioning, balance and coordination training, and strength exercises Diet and Exercise: Same SHD and MT program	Monthly low-intensity social activity session and general nutrition information; weekly phone contact; no structured diet or exercise program	SHD group: No significant change in HDL-C, calcium, or glucose MT Group: Significant decrease in glucose *p* = 0.025; No significant changes in HDL-C, calcium, or citrate SHD and MT Group: Significant decrease in HDL-C(*p* = 0.022)	Not reported	12 weeks
Xie et al., China [63]	Systematic review and network meta-analysis; 78 randomized controlled trials included; Total participants = 5219; Compared four combined interventions: Calorie restriction and exercise (CR + EX), time-restricted eating and exercise (TRF + EX), 5:2 intermittent fasting and exercise (5/2F + EX), ketogenic diet and exercise (KD + EX)	No major chronic disease; Included normal weight, overweight, and obese individuals; Aged 18–65 years	CR + EX: 15–30% calorie restriction combined with aerobic, resistance, or mixed exercise TRF + EX: Eating window typically 8 h with resistance or aerobic training 5/2F + EX: 2 fasting days per week with regular training KD + EX: very low-carbohydrate, high-fat ketogenic diet combined with exercise training	Exercise alone, dietary intervention alone, or no intervention	CR + EX: highly ranked for reductions in body weight, BMI, and body fat percentage. Relative effects varied across TRF + EX, 5/2F + EX, and KD + EX, indicating that body-composition responses differed according to the specific diet–exercise combination. TRF + EX: Moderate weight loss effect (SMD 2.37, 95% CI − 0.40 to 5.15 vs. CR + EX); Trend toward increased BMI (SMD 2.20, 95% CI 1.08 to 3.32) and body fat percentage (SMD 2.84, 95% CI 0.56 to 5.13) 5/2F + EX: No significant weight reduction vs. exercise alone (SMD 2.94, 95% CI − 3.64 to 9.52); Moderate BMI reduction effect (SMD 1.95, 95% CI − 0.49 to 4.39); Body composition changes less pronounced (SMD 2.66, 95% CI − 1.56 to 6.89) KD + EX: Smallest weight loss effect (SMD 1.80, 95% CI − 1.75 to 5.34 vs. CR + EX); Trend toward increased body fat percentage (SMD 3.14, 95% CI 0.52 to 5.75)	Not reported	4 weeks to 12 months
Dai et al., China [25]	Systematic review and meta-analysis; 12 randomized controlled trials; 616 participants(Age 40 ± 9 years; BMI 33.6 ± 4.8 kg/m²; 87.3% female) Compared intermittent fasting(IF) alone, intermittent fasting combined with exercise(EX)	Overweight or obesity adults; Age range 30–50 years; BMI ~ 33 kg/m²; Majority female participants; No major chronic diseases were reported across the included trials	IF types included: Time-restricted eating, alternate-day fasting, 5:2 intermittent fasting, ramadan intermittent fasting Exercise components: Aerobic exercise, resistance training, high-intensity interval training * Meta-analysis examined the combined effect of IF+exercise, not individual trial protocols separately	Intermittent fasting alone without added exercise component	IF + EX: Superior body composition improvements with greater fat mass reduction (−0.93 kg, 95% CI −1.69 to −0.18, *p* = 0.01) and waist circumference decrease (−2.51 cm, 95% CI −3.70 to −1.32, *p* < 0.001); Enhanced insulin sensitivity with lower insulin (−3.10 uIU/mL, 95% CI −4.25 to −1.95, *p* < 0.001) and HOMA-IR (SMD − 0.57, 95% CI −0.83 to −0.31, *p* < 0.001); Improved cardiorespiratory fitness (VO₂max + 1.92 mL/kg/min, 95% CI 0.32 to 3.52, *p* = 0.036) and lower resting heart rate (−2.68 bpm, 95% CI −4.71 to −0.64, *p* = 0.01); LDL cholesterol reduction (−10.67 mg/dL, 95% CI −20.00 to −1.35, *p* = 0.02) IF Alone: No significant differences in total body mass, BMI, or fat-free mass preservation; No blood pressure benefits compared to IF + EX; Less pronounced metabolic improvements; No cardiovascular fitness gains; Similar glucose control but inferior insulin sensitivity	Not reported	4 to 16 weeks
Sood et al., Australia [35]	Systematic review and meta-analysis; 20 papers from 17 randomized controlled trials	Cardiovascular disease, type 2 diabetes, metabolic syndrome, metabolic dysfunction–associated fatty liver disease, obesity, overweight, rheumatoid arthritis, colorectal cancer, prostate cancer, and breast cancer; Sample sizes per study ranged from 23 to 1521 participants; Aged ≥ 18 years	All Mediterranean diet interventions: All studies applying a Mediterranean diet, with or without caloric restriction or exercise * Mediterranean diet (MedDiet): High intake of vegetables, fruits, wholegrains, legumes, nuts, olive oil; moderate fish/dairy; reduced red/processed meat; Hypocaloric MedDiet: 500–950 kcal/day deficit, or ~ 30% caloric restriction; Isocaloric MedDiet: No energy restriction * Exercise intervention: Aerobic exercise(walking 30–45 min/day, 5–6 days/week), resistance training, combined aerobic and resistance training, step-based exercise(10,000 steps/day), or HIIT (4 × 4 min at 85–95% HRmax)	Usual diet, standard care dietary guidelines, or no structured exercise	MedDiet and Exercise: Most comprehensive body composition benefits with weight reduction (SMD − 0.19, ~ 2.5 kg absolute decrease, *p* = 0.01); BMI decrease (SMD − 0.27, ~ 1.0 kg/m² reduction, *p* < 0.001); Waist circumference reduction (SMD − 0.39, ~ 3.5 cm decrease, *p* = 0.04); Body fat and visceral fat decreased significantly (*p* = 0.01 and *p* = 0.02); Lean mass preserved (*p* = 0.76) Hypocaloric MedDiet and Exercise: Moderate effects on BMI (SMD − 0.24, *p* = 0.01), body fat (SMD − 0.13, *p* = 0.01), and visceral adiposity (SMD − 0.12, *p* = 0.02); No significant changes in weight, waist circumference, or lean mass; Less comprehensive than combined intervention Isocaloric MedDiet and Exercise: Significant weight (SMD − 0.18, *p* = 0.04) and BMI reduction (SMD − 0.24, *p* = 0.02); Notable waist circumference decrease (SMD − 0.32, *p* < 0.001); No significant body fat or lean mass changes; Demonstrates exercise benefits even without caloric restriction	Not reported	2 months to 3 years

*5/2F* 5:2 intermittent fasting, *BMI* body mass index, *BP* blood pressure, *CR* caloric restriction, *CREX* caloric restriction plus exercise, *CVD* cardiovascular disease, *DASH* dietary approaches to stop hypertension, *DASH-A* DASH diet alone, *DASH-WM* DASH diet plus weight management, *DBP* diastolic blood pressure, *EX* exercise, *HbA1c* glycated hemoglobin, *HDL-C* high-density lipoprotein cholesterol, *HIIT* high-intensity interval training, *HOMA-IR* homeostatic model assessment of insulin resistance, *HR* heart rate, *HRmax* maximal heart rate, *IF* intermittent fasting, *KD* ketogenic diet, *LDL-C* low-density lipoprotein cholesterol, *LV* left ventricular, *MedDiet* mediterranean diet, *MT* multicomponent training, *NS* not significant, *OGTT* oral glucose tolerance test, *SBP* systolic blood pressure, *SHD* sustainable healthy diet, *SMD* standardized mean difference, *TRE* time-restricted eating, *TREHIIT* time-restricted eating plus high-intensity interval training, *TRF* time-restricted feeding, *VO₂max* maximal oxygen uptake, *VO₂peak* peak oxygen uptake

The advantages of combining exercise with dietary restriction become more evident when examining glucose and lipid metabolism. Diet and exercise combination amplifies the effects of TRE alone on glucose and lipid metabolism indicators. Compared to TRE alone, combined interventions showed greater reductions in fasting insulin (−3.1µIU/ml, 95% CI: −4.25 to −1.95), HOMA-IR (−0.57, 95% CI: −0.83 to −0.31), LDL cholesterol (−10.67 mg/dL, 95% CI: −20 to −1.35), and significantly reduced resting heart rate (−2.68 bpm, 95% CI: −4.71 to −0.64) [25]. Combined exercise and TRE showed a −3.00mmHg reduction in systolic blood pressure compared to TRE alone (95% CI: −6.06 to 0.05, *p* = 0.05) [25]. Similarly, Apekey et al. (2011) found that groups combining CR with moderate exercise twice weekly showed significant reductions in blood pressure, with systolic (− 13 mmHg, *p* ≤ 0.05) and diastolic (− 8 mmHg, *p* ≤ 0.05) decreases that were not observed in the diet-only group [28].

Functional capacity represents the domain in which the advantages of combined interventions are most pronounced. Energy restriction alone produces little to no improvement in VO₂max and may even worsen cardiorespiratory fitness in some cases due to reductions in lean mass and overall energy availability (−0.1mL/kg/min). However, combining CR with exercise improved VO₂max by + 2.6mL/kg/min (approximately + 11%) [29]. While TRE alone showed no significant changes in VO₂peak, TRE combined with HIIT increased VO₂peak by + 3.2mL/kg/min (*p* < 0.05) [30]. Meta-analysis also confirmed that TRE plus exercise combinations significantly improved VO₂max compared to single intervention (+ 1.92mL/kg/min, 95% CI 0.32–3.52, *p* = 0.036) [25], suggesting that exercise combination comprehensively improves physiological reserve capacity.

Despite these clear benefits, some risk factors particularly blood pressure show inconsistent responses to energy-restriction strategies even when combined with exercise. These mixed effects suggest that focusing solely on caloric intake or timing is insufficient for comprehensive cardiometabolic risk management. As a result, integrating nutrient quality–focused dietary patterns (e.g., DASH, Mediterranean, plant-based diets) becomes essential, particularly for hypertension and lipid management, where food composition exerts strong effects independent of energy deficit.

## Synergistic Benefits of Nutrient Quality–Focused Dietary Approaches Combined with Exercise

The combination of the DASH diet and exercise demonstrates effects surpassing single interventions through the interaction of independent blood pressure reduction. In the ENCORE trial, participants assigned to DASH plus exercise achieved significantly greater reductions in blood pressure than those assigned to DASH alone (−16.1/- 9.9 mmHg, *p* < 0.001). Notably, vascular function improvements were observed only in the combined intervention group: pulse wave velocity reduction (*p* = 0.001), 33% increase in baroreflex sensitivity (*p* = 0.01), and left ventricular mass index reduction (*p* = 0.02), demonstrating that exercise induces vascular remodeling and vascular resilience unachievable through diet alone [31].

In a 12-month RCT, participants following the Mediterranean diet combined with exercise showed greater fasting glucose reduction (6.9 ± 0.8 to 5.9 ± 0.7mmol/L, *p* < 0.001) compared to controls (6.8 ± 0.9 to 6.4 ± 0.8mmol/L, *p* = 0.026) [32]. Similarly, Hernando-Redondo et al. (2020) reported that low-calorie Mediterranean diet combined with exercise significantly reduced fasting insulin (16.4 to 12.2µU/mL) and HOMA-IR (4.1 to 2.9, both *p* < 0.05) after 12 months [33]. Similar patterns are observed in plant-based diets. The GEICO trial showed that a low-fat vegan diet reduced HbA1c from 7.1% to 6.8% (−0.3%, *p* < 0.05) in overweight and type 2 diabetes patients [34].

Since nutrient quality-focused diets do not inherently target direct caloric restriction, weight loss effects remain limited even when combined with exercise. However, the Mediterranean diet with exercise showed significantly greater weight loss effects of −4.01 kg (95% CI: −5.79, −2.23 kg), compared to diet-only interventions (−1.75 kg) [22]. Recent meta-analysis of 20 RCTs also showed that Mediterranean diet combined with exercise demonstrated consistent improvements in body composition including body weight (−2.5 kg), BMI (−1.0 kg/m²), waist circumference (−3.5 cm), and visceral adipose tissue (−102 g) [35].

The combination of nutrient quality-focused diets and exercise shows complex and sometimes inconsistent patterns in lipid metabolism. Sampaio et al. (2024) demonstrated that the Mediterranean diet with exercise significantly increased unsaturated lipid levels, showing positive changes in lipid metabolism [36]. While HDL cholesterol shows consistent improvement in combined interventions, changes in LDL cholesterol and total cholesterol were limited or inconsistent. In the ENCORE study, the DASH plus exercise group showed a significant increase in HDL cholesterol (+ 4.2 mg/dL, *p* < 0.05). Still, LDL cholesterol reduction did not reach statistical significance (−8.1 mg/dL, *p* = 0.08) [37]. Combined lifestyle interventions may achieve partial cardiometabolic protection through improvements in HDL quality and increases in cardiovascular-protective fatty acids, but may require additional interventions to improve LDL and overall lipid profiles comprehensively.

When it comes to functional capacity, meaningful improvements are observed only with combined diet and exercise interventions. In a study of 144 overweight and hypertensive adults, the DASH diet combined with exercise resulted in a 19% increase in VO₂peak from baseline, reaching 29mL/kg/min. In contrast, DASH alone (−1.2%) and usual care (−3.2%) groups decreased (*p* < 0.001) [31]. Physical function outcomes also improved only in the combined intervention group, with significant increases in muscular endurance (push-ups: +5.4 reps, *p* < 0.05), grip strength (+ 2.2 kg, *p* < 0.05), and concurrent waist circumference reduction (*p* < 0.05) [38]. This suggests that exercise serves as the core driver of cardiopulmonary function and strength improvements, while the DASH diet’s antioxidant nutrients and electrolytes may amplify these effects through synergistic mechanisms.

Evidence indicates that nutrient-based dietary patterns, such as DASH, Mediterranean, and plant-based, confer unique and synergistic benefits across cardiometabolic and functional domains when integrated with exercise. Exercise not only amplifies dietary effects but also enables functional improvements, including aerobic capacity and muscle strength that are unachievable through diet alone. Particularly for patients with existing cardiometabolic disease or those with hypertension as a primary risk factor, the combination of the DASH diet and exercise may represent the primary recommended approach. These combined interventions can serve as comprehensive strategies to reduce cardiometabolic risk and optimize functional health.

## Implementation Features and Challenges of Diet Interventions

Dietary interventions provide several implementation advantages distinct from exercise, particularly in their accessibility, scalability, and minimal physical demands. Unlike exercise prescriptions, dietary changes can often be implemented without requiring specialized equipment, dedicated facilities, or high baseline physical capacity, making them feasible for patients with severe mobility restrictions, advanced frailty, or acute recovery periods [39–41]. Dietary interventions can be integrated into daily routines without scheduling specific “sessions,” allowing for continuous, flexible application. Recommendations can be tailored to cultural food preferences, religious practices, and individual tastes, thereby enhancing adherence when properly personalized. Dietary changes also enable broad metabolic impact, affecting weight, lipid profiles, glycemic control, and blood pressure, through a single lifestyle domain, and can be initiated rapidly in acute care or inpatient settings.

Despite these advantages, sustaining long-term dietary change requires consistent access to appropriate foods, which can be limited by cost, individual food environments, or seasonal availability. Cultural traditions, family eating patterns, and social settings can either support or undermine adherence, sometimes making it difficult to maintain prescribed diets over the long term [42, 43]. Nutrition literacy and cooking skills vary widely; patients without these skills may struggle to translate dietary advice into actionable meals [44–46]. Physiological benefits such as weight loss or lipid improvements often take weeks to months to manifest, which may diminish early motivation compared to interventions with more immediate perceptible effects. For some individuals, restrictive diets can reduce quality of life, create perceptions of social isolation, or trigger disordered eating behaviors, all of which can impair adherence [47]. Unlike exercise interventions, where heart rate, repetitions, or distances can be objectively tracked, dietary intake is often self-reported and subject to recall bias, underreporting, or intentional misrepresentation.

## Implementation Features and Challenges of Exercise Interventions

Exercise interventions offer several features that make them particularly amenable to personalization and long-term adherence. Unlike dietary prescriptions, which are often communicated as static recommendations, exercise interventions typically involve real-time, face-to-face interaction between patient and provider. This direct supervision enables immediate feedback on safety, technique, and performance, while also allowing dynamic adjustment of intensity, volume, and modality in response to the individual’s daily health status, fatigue, or recovery capacity. Exercise programs are also highly adaptable to personal preferences, whether through walking, cycling, resistance training, or aquatic modalities, which enhances enjoyment, intrinsic motivation, and adherence. In-session monitoring of heart rate, perceived exertion, and movement form further ensures safety, particularly for patients with cardiovascular disease, frailty, or multiple comorbidities [48, 49]. Beyond their physiological effects, exercise sessions function as therapeutic opportunities that foster patient–provider rapport, build confidence, and support continuous skill acquisition, promoting independent self-management and long-term lifestyle integration [50, 51].

Despite their advantages, logistical and ecological obstacles are common in community and outpatient contexts. Patients often encounter difficulties with transportation to supervised programs, limited access to safe and appropriate exercise spaces, and the financial burden of program costs, all of which constrain regular participation [52–54]. Medical limitations further complicate adherence. Comorbid conditions such as severe arthritis or advanced heart failure, as well as treatment-related fatigue in clinical populations, can reduce exercise tolerance and delay progression toward target intensities [55–57]. Low self-efficacy, fear of injury or symptom exacerbation, and negative past experiences with exercise can undermine adherence, even when structured programs are readily available. Importantly, patients may perceive fewer immediate benefits from exercise compared to medications or dietary changes, diminishing motivation for sustained engagement [58–60]. Safe progression requires careful adjustment of intensity, volume, and modality according to fluctuating health status. Without such personalization and supervision, dropout rates increase and the durability of long-term benefits is significantly reduced.

## Future Directions & Translational Perspectives

Equally important is the need for integrated care pathways that deliver exercise and dietary counseling within the same clinical system rather than as separate, uncoordinated services. For example, cardiac rehabilitation programs could incorporate structured nutrition goals alongside individualized exercise prescriptions, allowing patients to receive both components as part of a unified treatment plan. Technology can amplify this integration by linking dietary logs with wearable-derived metrics, enabling real-time feedback and dynamic adjustment of caloric intake or exercise dose. Long-term sustainability must also be prioritized: structured maintenance phases with reduced-frequency supervision, periodic booster sessions, and community-based peer networks may help preserve benefits beyond the typical trial window. Finally, ensuring equity requires adapting interventions to cultural dietary norms, improving access to healthy foods and safe activity spaces, and delivering programs through telehealth in underserved regions. Collectively, these strategies move beyond theoretical synergy to outline concrete, scalable pathways for translating combined lifestyle interventions into durable population-level reductions in cardiovascular disease burden.

Future efforts should shift from uniform recommendations to adaptive strategies that specify when and how diet and/or exercise should be deployed. Rather than promoting simultaneous initiation in all patients, sequencing approaches may prove more effective. For example, frail or sarcopenic patients may benefit from prioritizing resistance training to preserve lean mass before introducing substantial caloric restriction, while those with severe obesity or uncontrolled diabetes may require early dietary restriction to reduce metabolic load before progressing to structured exercise. Similarly, phenotype-guided tailoring is essential. Patients with dyslipidemia may respond best to Mediterranean or plant-based diets, whereas those with impaired mobility or low cardiorespiratory reserve may benefit from exercise-centered prescriptions. Importantly, the clinical value of exercise extends beyond changes in body weight and conventional cardiometabolic risk factors. Physical activity and, particularly, cardiorespiratory fitness are consistently associated with lower cardiovascular and all-cause mortality, while cardiorespiratory fitness provides additional prognostic information by reflecting integrated multisystem function and physiological reserve [61, 62]. These strategies should be evaluated in randomized trials that stratify by baseline clinical profiles, thereby moving lifestyle medicine toward genuine precision care.

## Conclusion

Diet and exercise remain central pillars in cardiometabolic disease prevention and management, yet their benefits are not equivalent. Energy restriction strategies demonstrate superior efficacy for weight reduction and lipid modification, while nutrient-quality interventions provide robust cardiovascular protection through anti-hypertensive and anti-inflammatory mechanisms. Exercise training uniquely enhances physical function and preserves lean mass. When combined, these interventions produce synergistic effects that exceed their individual contributions, with exercise amplifying dietary benefits while preventing lean mass loss and optimizing body composition. While the beneficial effects of combined diet and exercise interventions are well documented, further research is needed to explore optimal intervention sequencing and patient-specific tailoring strategies. To date, most studies examining these relationships have short follow-up periods and limited diversity in study populations, and few have investigated the long-term sustainability of combined interventions.

Recent evidence has begun to illuminate the synergistic potential of multimodal approaches, indicating that a strategic combination of energy restriction, nutrient optimization, and exercise training may produce superior cardiovascular outcomes compared to single-modality interventions. However, these findings require validation in larger, more diverse cohorts with extended follow-up periods. Future research should prioritize the development of phenotype-guided intervention protocols, examination of optimal sequencing strategies, and evaluation of technology-enhanced delivery models. Accessibility, adherence, and healthcare integration remain significant barriers, underscoring the need for innovative approaches that integrate diet and exercise prescriptions into unified care models. Ultimately, the right strategy delivered to the right patient at the right time is key to maximizing both immediate therapeutic benefits and long-term cardiovascular health outcomes across diverse populations.

## Key References

- Dai Z-H, et al. Additional Effect of Exercise to Intermittent Fasting on Body Composition and Cardiometabolic Health in Adults With Overweight/obesity: A Systematic Review and Meta-analysis. Current Obesity Reports. 2025;14(1):54.
  - ○ This meta-analysis directly addresses the central question of this review by evaluating the additional effects of exercise when combined with intermittent fasting. The combined intervention improved selected body composition and cardiometabolic outcomes compared with intermittent fasting alone, while several other outcomes showed no significant additional benefit, highlighting the outcome-specific nature of combined lifestyle interventions.
- Sood S, et al. The effects of Mediterranean diet with and without exercise on body composition in adults with chronic disease: A systematic review and meta-analysis of clinical trials. Clinical Nutrition. 2025.
  - ○ This systematic review and meta-analysis provides contemporary evidence on Mediterranean diet interventions with and without exercise in adults with chronic disease. Mediterranean diet plus exercise was associated with improvements across multiple body composition outcomes, supporting the potential value of a multimodal lifestyle approach while demonstrating that the magnitude and pattern of benefit vary by outcome.
- Xie Y, Gu Y, Li Z, He B, Zhang L. Effects of Different Exercises Combined with Different Dietary Interventions on Body Composition: A Systematic Review and Network Meta-Analysis. Nutrients. 2024;16(17):3007.
  - ○ This network meta-analysis compares multiple combinations of dietary and exercise interventions, providing a broader framework for evaluating their relative effects on body composition. The findings reinforce that the effectiveness of combined lifestyle interventions depends on the specific diet–exercise pairing and the outcome being targeted.

## Data Availability

No datasets were generated or analysed during the current study.
